# Diet Quality, Healthy Practices, and Psychosocial Functioning Across School Youth, Students, and Adults in Poland: A Cross-Sectional Online Survey

**DOI:** 10.3390/nu18122022

**Published:** 2026-06-21

**Authors:** Klaudia Sochacka, Agata Kotowska, Sabina Lachowicz-Wiśniewska

**Affiliations:** 1 Doctoral School, The Faculty of Medicine and Health Science, University of Kalisz, W. Bogusławskiego 2, 62-800 Kalisz, Poland; szd.5.2023@uniwersytetkaliski.edu.pl; 2 Institute of Sociology, University of Rzeszów, 35-959 Rzeszów, Poland; akotowska@ur.edu.pl; 3 Department of Nutrition and Food, The Faculty of Medicine and Health Science, University of Kalisz, W. Bogusławskiego 2, 62-800 Kalisz, Poland; 4 Department of Biotechnology and Food Analysis, Wroclaw University of Economics and Business, Komandorska 118/120 53-345 Wroclaw, Poland

**Keywords:** diet quality, healthy practices, obesity knowledge, depression knowledge, psychosocial well-being, stress/overload, school youth, students, adults

## Abstract

**Background:** This study aimed to compare a limited set of predefined diet-, lifestyle-, knowledge-, and psychosocial indicators across school youth, students, and adults in Poland, and to examine their associations with three predefined outcomes: BMI ≥ 25 kg/m^2^, poorer mental well-being, and high stress/overload. Diet quality, daily health-related practices, psychosocial well-being, and stress/overload may co-occur across different life stages, but online survey data require a focused analytical framework to avoid overinterpretation. **Methods:** This cross-sectional anonymous online survey included 360 respondents: 154 school youth aged 15–19 years, 127 students aged 20–29 years, and 79 adults aged 30 years or older. Dietary assessment was based on the KomPAN questionnaire and included the pro-healthy diet index, non-healthy diet index, and Diet Quality Index. Study-specific scores were used for knowledge, healthy practices, psychosocial well-being, and stress/overload. Analyses were restricted to predefined group comparisons, selected correlations, and three whole-sample adjusted logistic regression models. **Results:** Adults had the highest BMI and waist/hip circumference, whereas school youth showed the highest non-healthy diet index and more frequent high processed-food intake. Among the knowledge and psychosocial indicators, only obesity knowledge differed significantly between groups, with the highest mean value among students. Stress/overload was inversely associated with psychosocial well-being, and DQI was positively associated with psychosocial well-being after adjustment for age, sex, and group. In adjusted whole-sample models, BMI ≥ 25 kg/m^2^ was positively associated with age and DQI and inversely associated with physical activity frequency and regular meals; the positive DQI–BMI association was interpreted cautiously as potentially reflecting reverse causality, reporting bias, or compensatory dietary modification among respondents with excess body weight. Poorer mental well-being was associated with higher stress/overload and inversely associated with DQI, physical activity frequency, and family meals. High stress/overload was positively associated with highly processed food intake and inversely associated with regular meals. **Conclusions:** The findings suggest that diet quality, behavioral regularity, and psychosocial burden may be more informative than knowledge alone when describing health-related profiles across age-defined groups. Because the study was cross-sectional, self-reported, anonymous, and based on a modest sample, the results should be interpreted as preliminary and hypothesis-generating rather than causal.

## 1. Introduction

Diet quality, physical activity, sleep, meal regularity, psychosocial well-being, and perceived stress are interrelated components of everyday health functioning. Their relevance is particularly evident during adolescence and early adulthood, when health-related habits are still being established, educational and social pressures are high, and many mental health problems first emerge. The World Health Organization reports that mental disorders affect a substantial proportion of adolescents, with anxiety and depression among the most important contributors to illness and disability in this age group [1,2]. At the population level, the second Lancet Commission on adolescent health and wellbeing also emphasizes the increasing co-occurrence of mental health burden, poor nutrition, and overweight/obesity among young people [3]. In adults, depression, obesity, and stress-related burdens continue to contribute substantially to impaired functioning, reduced quality of life, and higher public health risk [2,4]. These observations justify studies that consider mental and metabolic health not as isolated domains, but as co-occurring dimensions of daily lifestyle and psychosocial functioning.

At the same time, the presence of health-related knowledge does not necessarily imply successful behavioral implementation. The knowledge-practice gap is a persistent challenge in public health: individuals may understand the importance of healthy eating, physical activity, or sleep but still face barriers related to time pressure, stress, social context, food environment, or emotional burden. Behavior-change frameworks indicate that knowledge is only one component of behavioral capability and is insufficient when opportunity, motivation, and environmental support are limited [5,6]. Recent Polish data among adolescents also show that nutrition-related knowledge and nutrition-related practice are related but not equivalent constructs, and that the implementation of dietary recommendations may remain substantially lower than declared knowledge [7]. In studies comparing different age-defined groups, it is therefore important to distinguish knowledge from actual practices and psychosocial resources.

Diet quality is one of the modifiable domains that may link metabolic and psychological functioning. Nutritional psychiatry has emphasized that dietary patterns, rather than single nutrients alone, may be relevant to mental health and emotional functioning [8]. Meta-analytic evidence from randomized controlled trials suggests that dietary improvement can reduce depressive symptoms, although the magnitude and clinical relevance of effects vary across populations and intervention designs [9]. In adolescents specifically, recent systematic review evidence indicates that healthier dietary patterns and higher diet quality are generally associated with more favorable mental health indicators, whereas poorer diet quality is more often linked to depressive symptoms, anxiety, or psychological distress [10]. Conversely, higher intake of ultra-processed foods has been associated with poorer mental-health outcomes in observational evidence, including depressive and anxiety symptoms [11]. These findings support the inclusion of diet-quality indicators when examining psychosocial well-being and stress-related outcomes.

Daily routines may be equally important. Irregular meals, insufficient sleep, and low physical activity can co-occur with stress and reduced self-regulation. Sleep duration and sleep quality are linked with cardiometabolic health and lifestyle behaviors, and insufficient sleep may affect appetite regulation, food reward, and cognitive control [12,13]. Physical activity is also recognized as a core health behavior across age groups, with international guidelines emphasizing its importance for physical and mental health [14,15]. From this perspective, meal regularity, sleep, and physical activity should be considered not merely as isolated lifestyle variables, but as routine-based behaviors that may shape both metabolic and psychosocial health profiles.

The gut–brain axis and inflammation-related pathways provide a biological framework for understanding why diet, stress, and psychosocial functioning may be connected. The microbiota–gut–brain axis involves bidirectional communication between the gastrointestinal tract and the central nervous system through immune, endocrine, neural, and metabolic routes [16]. Recent reviews focusing on diet quality, anxiety, and youth mental health suggest that gut microbiome-related mechanisms may be relevant to psychological outcomes, but the available evidence remains preliminary and heterogeneous [17,18]. Therefore, such mechanisms should not be interpreted as directly demonstrated by the present survey data. Rather, they provide a cautious conceptual background for investigating the co-occurrence of diet quality, stress/overload, and psychosocial well-being.

School youth, students, and adults may differ in how diet quality, routines, stress, and knowledge are distributed. School youth are exposed to school-related stress and family-dependent food environments, students often experience transitions toward independent living and irregular routines, and adults may face occupational, family, and metabolic burdens. However, many cross-sectional studies either focus on single age groups or examine diet, physical activity, knowledge, and mental well-being separately. Our previous work in Polish adolescents and young adults showed suboptimal diet quality and nutrition misconceptions, while the present study extends this perspective by examining a broader but deliberately limited set of diet-, lifestyle-, knowledge-, and psychosocial indicators across three age-defined groups [19].

The revised aim of this study was to examine whether three age-defined groups in Poland—school youth, students, and adults—differ in a limited set of predefined anthropometric, diet-quality, lifestyle, knowledge, and psychosocial indicators, and whether these indicators are associated with three predefined health-related outcomes: BMI ≥ 25 kg/m^2^, poorer mental well-being, and high stress/overload. The study was guided by three research questions: (RQ1) Do the three groups differ in selected predefined health-related indicators? (RQ2) Are diet quality, healthy practices, psychosocial well-being, and stress/overload associated with one another in the total sample after accounting for multiple testing? (RQ3) Which predefined dietary, behavioral, and psychosocial variables are independently associated with BMI ≥ 25 kg/m^2^, poorer mental well-being, and high stress/overload in whole-sample adjusted models?

## 2. Materials and Methods

### 2.1. Study Design and Participants

This cross-sectional study was conducted in Poland using an anonymous online survey designed to assess anthropometric status, diet quality, lifestyle behaviors, knowledge related to obesity and depression, healthy practices, psychosocial well-being, and perceived stress/overload across different age-defined groups. Data were collected between December 2025 and March 2026 through web-based dissemination of the questionnaire via institutional channels, social media, and peer-to-peer sharing.

The study included respondents aged 15 years and older who resided in Poland and provided informed consent to participate. For participants younger than 18 years, assent and parental/legal guardian consent were obtained in accordance with applicable ethical requirements. Responses that were incomplete or contained implausible anthropometric values were excluded from the final dataset.

For descriptive analyses, the final study sample comprised 360 respondents, including 154 school youth aged 15–19 years, 127 students aged 20–29 years, and 79 adults aged 30 years or older. Model-specific sample sizes are reported in the corresponding tables where applicable. To reduce duplicate entries, the survey platform restricted multiple submissions from the same device/IP where feasible, and response timestamps were screened for improbably short completion times.

The study protocol received approval from the Bioethics Committee of the University of Kalisz (Approval No. 20/2025) and was conducted in accordance with the ethical standards of the Declaration of Helsinki [20]. Recruitment commenced only after ethical approval had been granted on 24 October 2025.

### 2.2. Survey Instrument

Data were collected using an author-designed questionnaire developed for the purposes of this study, with dietary assessment based on the KomPAN [21,22,23] dietary habits and nutrition beliefs questionnaire and additional modules addressing knowledge, health-related practices, psychosocial functioning, and stress-related experiences. The survey was administered online in Polish.

The questionnaire covered the following domains: sociodemographic characteristics, self-reported anthropometric data, dietary habits and food-frequency intake, health-related knowledge, healthy daily practices, psychosocial well-being, perceived stress/overload, and stress-coping strategies. A detailed overview of the questionnaire domains, item direction, scoring rules, and construction of composite variables is provided in Supplementary Table S1.

Self-reported anthropometric variables included body weight, height, and waist/hip circumference. Body mass index (BMI, kg/m^2^) was calculated from reported body weight and height. In selected analyses, excess body weight was defined as BMI ≥ 25 kg/m^2^ and obesity as BMI ≥ 30 kg/m^2^.

Dietary intake was assessed using a food-frequency module based on the KomPAN questionnaire. Food-frequency categories were converted into standardized daily-frequency values according to the KomPAN manual. The pro-healthy diet index (pHDI) was calculated from predefined pro-healthy food groups, whereas the non-healthy diet index (nHDI) was calculated from predefined less favorable food groups. Both indices were expressed on a 0–100 scale. The Diet Quality Index was calculated as DQI = pHDI − nHDI; therefore, its theoretical range was −100 to +100, with higher values indicating a more favorable balance between pro-healthy and non-healthy dietary components. The food groups included in pHDI and nHDI are listed in Supplementary Table S1.

Knowledge-related variables were calculated separately for obesity-related and depression-related knowledge. Correct responses were coded as 1, whereas incorrect or “do not know” responses were coded as 0. Domain-specific scores were calculated and additionally summarized as an overall knowledge score. The healthy practices score was derived from items describing routine health-related behaviors, with more favorable responses assigned higher values. The psychosocial well-being score was derived from items describing positive psychosocial functioning, with higher values indicating better well-being. The stress/overload score was derived from items reflecting perceived stress, overload, and related burden, with higher values indicating greater burden. Where necessary, negatively worded items were reverse-coded so that all items within each score followed the same direction.

The knowledge–practice gap indicator was calculated as the difference between the standardized overall knowledge score and the standardized healthy practices score, with higher values indicating a greater discrepancy between declared knowledge and reported implementation. Detailed item composition, coding direction, score range, and interpretation of all author-derived composite variables are provided in Supplementary Table S1.

Lifestyle variables included self-rated physical activity, frequency of physical activity, sleep duration, regularity of meals, frequency of family meals, frequency of home-prepared meals, and intake of highly processed food. For selected analyses, binary indicators were created, including short sleep duration (<7 h/night), low physical activity, high processed-food intake, low fruit-and-vegetable intake, low family-meal frequency, poor diet quality defined as the lowest DQI tertile, and high knowledge–practice gap. The coding rules and analytical thresholds used to construct these binary variables are provided in Supplementary Table S2.

Stress-related supplementary analyses were based on two complementary components. First, respondents indicated major sources of stress, including work/job demands, family/personal problems, financial problems, school/exam pressures, and health-related stress. Second, they reported their preferred coping strategies, including spending time with family or friends, physical exercise, meditation/yoga, music/reading/relaxation, sleep/rest, professional support, and non-adaptive coping. These variables were treated as contextual supplementary indicators.

Before final administration, the questionnaire was reviewed for clarity and wording. Because the questionnaire combined heterogeneous constructs, including knowledge, practices, psychosocial well-being, and stress/overload, no single global Cronbach’s alpha was interpreted for the entire instrument. Instead, the construction, coding direction, and interpretation of each author-derived composite variable are reported separately in Supplementary Table S1.

### 2.3. Statistical Analysis

The statistical analysis plan was revised to reduce the risk of overinterpretation and to align the analytical scope with the cross-sectional design and sample size. Three main outcomes were predefined: (1) BMI ≥ 25 kg/m^2^ among participants aged 18 years or older, (2) poorer mental well-being, and (3) high stress/overload, defined as the upper tertile of the stress/overload distribution. The main explanatory variables were limited to predefined diet-quality, lifestyle, knowledge-related, and psychosocial indicators. For the BMI ≥ 25 kg/m^2^ model, the analytical sample was restricted to respondents aged ≥18 years because adult BMI cut-offs for excess body weight were applied. Although the descriptive sample included respondents aged 15–19 years, 36 participants were younger than 18 years and were therefore excluded from this model. Consequently, the BMI ≥ 25 kg/m^2^ logistic regression model included 324 respondents: 118 school-youth respondents aged 18–19 years, 127 students aged 20–29 years, and 79 adults aged ≥30 years.

Continuous variables are presented as mean ± standard deviation, and categorical variables as N (%). Between-group comparisons across school youth, students, and adults were conducted using ANOVA or Kruskal–Wallis tests for continuous or ordinal-type variables and chi-square or Fisher exact tests for categorical variables, as appropriate [24]. Effect sizes were reported as η^2^ for continuous outcomes and Cramér’s V for categorical outcomes.

To reduce the risk of overinterpretation related to multiple testing, the analytical scope was narrowed to predefined comparisons and selected whole-sample associations. Between-group comparisons were interpreted primarily with reference to effect sizes and the consistency of findings rather than isolated nominal *p*-values. Benjamini–Hochberg false discovery rate (FDR) correction was applied to the predefined correlation analyses, for which both unadjusted *p*-values and FDR-adjusted q-values are reported. For multivariable models, interpretation was based on adjusted odds ratios, 95% confidence intervals, model fit, and conceptual consistency.

Associations among the main analytical variables were assessed using Spearman’s rank correlation coefficient. Partial correlations adjusted for age, sex, and study group were calculated for selected relationships central to the research questions.

Multivariable logistic regression models were fitted for the three predefined outcomes. Covariates were selected a priori based on the conceptual framework and were limited to reduce the risk of overfitting. Model-specific complete-case analysis was used, and the number of observations included in each model. Adjusted odds ratios with 95% confidence intervals were reported. Multicollinearity among predictors was assessed using variance inflation factors. All VIF values were low, with maximum VIF values of 1.443, 1.402, and 1.356 in the BMI ≥ 25 kg/m^2^, poorer mental well-being, and high stress/overload models, respectively, indicating no problematic multicollinearity.

PCA was performed on standardized KomPAN food-frequency variables using principal component extraction. The suitability of the data for PCA was assessed using the Kaiser–Meyer–Olkin measure of sampling adequacy and Bartlett’s test of sphericity. The overall KMO value was 0.699, and Bartlett’s test was significant (χ^2^ = 2930.20, *df* = 300, *p* < 0.001), indicating that the correlation matrix was suitable for exploratory dimension reduction. Components retained for supplementary description were selected based on scree-plot inspection, explained variance, coherent loading structure, and interpretability. The retained five components explained 50.43% of the total variance. Loadings with an absolute value ≥ 0.30 were considered informative. No rotation was applied because PCA was used only for supplementary descriptive dimension reduction. PCA findings were treated as exploratory and were not used as a basis for confirmatory conclusions.

Clustering was conducted using k-means clustering with Euclidean distance on z-standardized variables, including BMI, DQI, overall knowledge, healthy practices, stress/overload, and psychosocial well-being. Candidate solutions from two to six clusters were compared using cluster-size distribution, interpretability, within-cluster sum of squares, silhouette coefficient, Calinski–Harabasz index, and Davies–Bouldin index. The three-cluster solution was retained because it provided interpretable, non-trivial profiles with acceptable cluster-size distribution and an improved Davies–Bouldin index compared with the two-cluster solution. Cluster analysis was considered exploratory and was not used for confirmatory inference.

A sensitivity power calculation was added for the final sample size. For a chi-square comparison of a binary outcome across three age-defined groups, N = 360 provided approximately 80% power at α = 0.05 to detect an effect of Cramér’s V 0.164. For a one-way ANOVA across three groups, the same sample size provided approximately 80% power to detect f 0.164, corresponding to η^2^ ≈ 0.026. These calculations indicate that the study was suitable for detecting small-to-moderate whole-sample group differences, but not small effects or stable subgroup-specific multivariable associations.

Descriptive statistics, between-group comparisons, correlation analyses, and logistic regression models were performed using Statistica 13.3. Benjamini–Hochberg FDR correction for the predefined correlation analyses, PCA diagnostics, cluster-validation indices, and VIF-based multicollinearity diagnostics were verified using Python 3.11. Microsoft Excel was used only for data organization and table preparation. The level of significance was set at *p* < 0.05, and all tests were two-sided.

## 3. Results

### 3.1. Study Sample and Predefined Between-Group Comparisons

The final analytical sample included 360 respondents: 154 school youth aged 15–19 years, 127 students aged 20–29 years, and 79 adults aged 30 years or older. Women constituted 63.6% of the total sample, although the sex distribution differed significantly across groups, with the lowest proportion of women among school youth and the highest among adults. Adults had the highest mean BMI and waist/hip circumference, whereas school youth had the lowest mean BMI. Mean body weight did not differ significantly between groups, while height, BMI, and waist/hip circumference showed significant between-group differences (Table 1).

Among the predefined diet-quality indicators, the non-healthy diet index (nHDI) differed significantly across the three age-defined groups, with the highest mean value observed in school youth and the lowest in adults. In contrast, the pro-healthy diet index (pHDI) and the overall Diet Quality Index (DQI) did not differ significantly between groups. This indicates that the main dietary contrast between groups was related primarily to the frequency of less favorable food choices rather than to global diet-quality score differences (Table 1).

Selected lifestyle indicators also differed across groups. High or very high self-rated physical activity and physical activity performed at least three times per week were most frequent among school youth and least frequent among adults. Highly processed food intake at least three times per week was more common among school youth and students than among adults. Sleep duration below 7 h/night was frequent in all three groups and did not differ significantly between them (Table 1 and Table 2).

Among the knowledge-related and psychosocial variables, only the obesity knowledge score differed significantly across groups, with the highest mean value observed among students. Depression knowledge, overall knowledge, healthy practices, psychosocial well-being, stress/overload, and the knowledge–practice gap did not show significant between-group differences. Therefore, knowledge-related differences were limited and were not accompanied by parallel between-group differences in the broader psychosocial or practice-related scores (Table 1).

### 3.2. Predefined Risk Outcomes Across Age-Defined Groups

**Figure 1:**
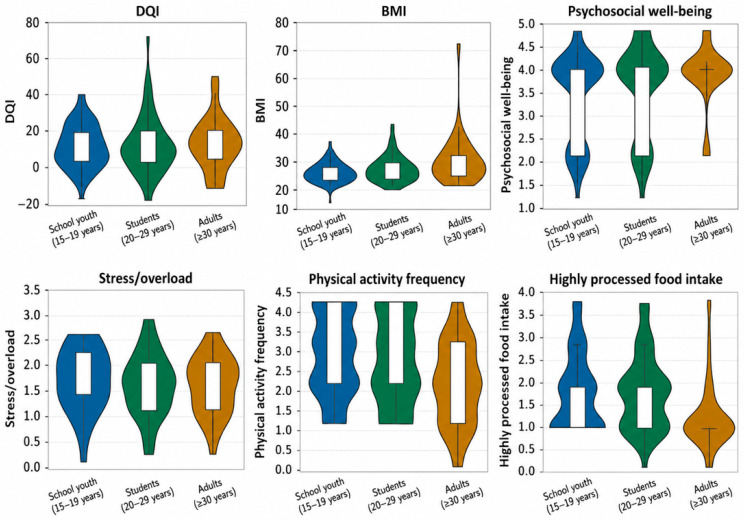
Distribution of selected predefined health-related indicators by age-defined group. Violin plots show the distribution of Diet Quality Index (DQI), body mass index (BMI), psychosocial well-being, stress/overload, physical activity frequency, and highly processed food intake among school youth aged 15–19 years, students aged 20–29 years, and adults aged ≥30 years.

The prevalence of BMI ≥ 25 kg/m^2^ differed significantly across the three groups and increased from school youth to students and adults. BMI ≥ 30 kg/m^2^ showed a similar age-related gradient. Poorer mental well-being was most frequent among school youth and least frequent among adults. High stress/overload was numerically most frequent among school youth, but the between-group difference did not reach statistical significance (Table 2; Figure 1).

Among the additional binary risk indicators, low physical activity was most frequent among adults, while high processed-food intake was more frequent among school youth and students than among adults. No significant between-group differences were observed for short sleep, poor diet quality defined as the lowest DQI tertile, low fruit-and-vegetable intake, low family-meal frequency, or high knowledge–practice gap. Overall, the most consistent between-group contrasts concerned anthropometric status, non-healthy dietary exposure, physical activity, and selected psychosocial risk outcomes (Table 2; Figure 1).

### 3.3. Associations Among Predefined Core Variables

In the total sample, stress/overload was inversely associated with psychosocial well-being and represented the strongest observed association among the predefined psychosocial variables. Healthy practices were positively associated with psychosocial well-being and inversely associated with stress/overload. Overall knowledge showed a weaker positive association with healthy practices (Table 3).

Among the diet-related variables, DQI was positively associated with psychosocial well-being. After adjustment for age, sex, and group, the positive association between DQI and psychosocial well-being remained evident, and DQI also showed a weak inverse association with stress/overload. BMI was inversely associated with healthy practices after adjustment, whereas no adjusted association was observed between BMI and DQI. These findings indicate that the core behavioral and psychosocial constructs were interrelated; however, the observed associations were generally modest and should be interpreted as cross-sectional co-occurrence patterns rather than causal relationships (Table 3).

### 3.4. Adjusted Models for the Three Predefined Outcomes

**Figure 2:**
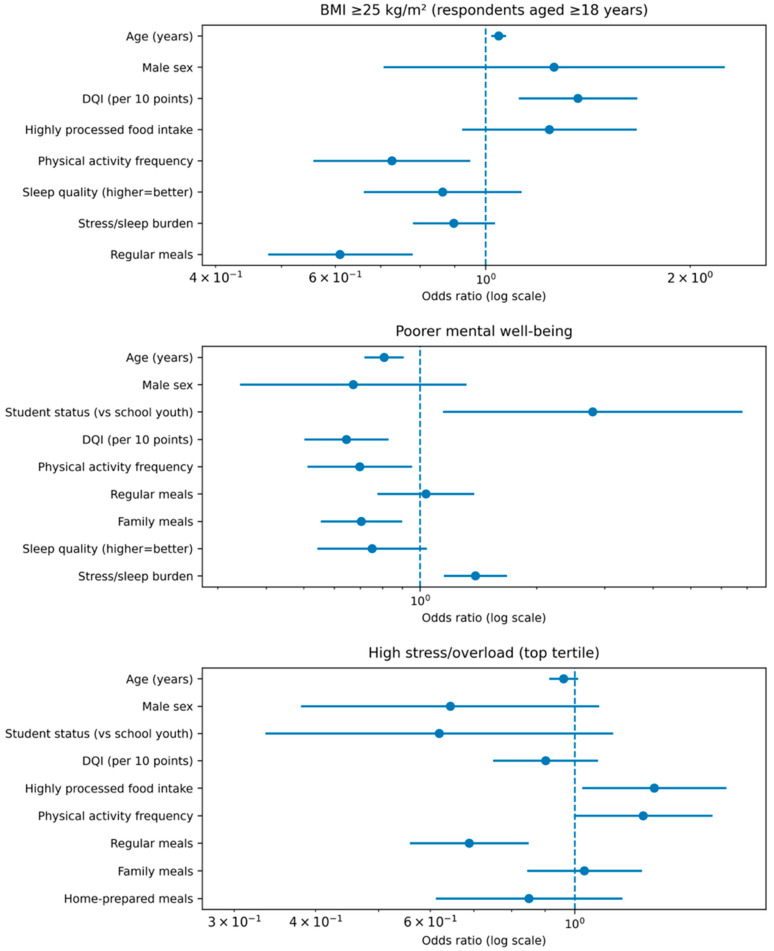
Adjusted associations with predefined outcomes.

In the adjusted model for BMI ≥ 25 kg/m^2^ among respondents aged 18 years and older, higher age and higher DQI were associated with greater odds of the outcome, whereas more frequent physical activity and more regular meals were associated with lower odds. The positive association between DQI and BMI ≥ 25 kg/m^2^ should be interpreted cautiously, because reverse causality, weight-related dietary modification, or reporting bias among individuals with excess body weight cannot be excluded (Table 4, Figure 2).

In the adjusted model for poorer mental well-being, higher stress/overload was associated with greater odds of the outcome. In contrast, higher age, higher DQI, more frequent physical activity, and more frequent family meals were associated with lower odds of poorer mental well-being. This model showed the strongest explanatory performance among the three predefined outcomes, suggesting that poorer mental well-being was more closely related to the combined psychosocial and behavioral profile than to knowledge-related variables alone (Table 4, Figure 2).

In the adjusted model for high stress/overload, higher intake of highly processed food was associated with greater odds of the outcome, whereas more regular meals were associated with lower odds. DQI, family meals, and home-prepared meals were not independently associated with high stress/overload after adjustment. Thus, high stress/overload appeared to be more closely related to selected routine-related and processed-food indicators than to the overall diet-quality score (Table 4, Figure 2).

Exploratory analyses, including stress-source/coping distributions, dietary-pattern analysis, and cluster analysis, are presented in the Tables S3–S5; Figures S1 and S2 were not used as a basis for confirmatory interpretation (Tables S3–S5; Figures S1 and S2).

## 4. Discussion

The present study examined predefined diet-related, lifestyle, knowledge-related, and psychosocial indicators across school youth, students, and adults in Poland, and assessed their associations with three predefined outcomes: BMI ≥ 25 kg/m^2^, poorer mental well-being, and high stress/overload. The revised analyses indicate that health-related knowledge and everyday health-related practices should not be treated as interchangeable constructs. Among the knowledge-related and psychosocial variables, only obesity-related knowledge differed significantly across the three age-defined groups, with the highest mean value observed among students. In contrast, depression knowledge, overall knowledge, healthy practices, psychosocial well-being, stress/overload, and the knowledge-practice gap did not differ significantly at the group-mean level. At the same time, adults had the highest BMI and waist/hip circumference, whereas school youth showed the highest non-healthy diet index and a less favorable profile for selected lifestyle-related indicators. These findings suggest that greater knowledge alone was not accompanied by a uniformly more favorable behavioral profile across life-stage groups. This interpretation is consistent with the view that cognitive awareness, although important, is often insufficient to produce sustained behavioral change when opportunity, motivation, environmental support, and routine-related conditions are limited [5,6,7].

A key dietary finding was that pHDI and overall DQI did not differ significantly between groups, whereas nHDI was highest among school youth and lowest among adults. This suggests that the main age-related dietary contrast was driven more by the frequency of less favorable food choices than by clear differences in the pro-healthy component of diet quality. This observation is relevant in the context of nutritional psychiatry, where poorer dietary patterns are increasingly considered in relation not only to metabolic health but also to psychological functioning [8,9]. In the present study, DQI was positively associated with psychosocial well-being in the total sample, and this association remained evident after adjustment for age, sex, and group. These results support the interpretation of diet quality as one component of a broader behavioral and psychosocial profile rather than as an isolated determinant. They are also broadly consistent with our previous findings in Polish adolescents and young adults, in whom overall diet quality was suboptimal and nutrition misconceptions were common, particularly in younger respondents [19].

At the same time, the association between DQI and BMI ≥ 25 kg/m^2^ requires careful interpretation. In the adjusted whole-sample model, higher DQI was associated with higher odds of BMI ≥ 25 kg/m^2^. This direction should not be interpreted causally. A more plausible explanation may involve reverse causality, reporting bias, or compensatory dietary modification, whereby respondents with excess body weight may have already introduced healthier eating behaviors or may report their diet more favorably because of weight-related concerns. Because this study was cross-sectional and based on self-reported dietary and anthropometric data, the observed association should not be understood as evidence that better diet quality promotes excess body weight. Rather, it may reflect the complexity of dietary self-management in individuals already aware of weight-related or metabolic risk. This interpretation is consistent with broader methodological concerns in observational nutrition research, where current diet quality may partly reflect responses to existing health status rather than its original cause [5,9].

The findings also highlight the relevance of psychosocial burden and everyday behavioral regularity. In the total sample, stress/overload showed the strongest inverse association with psychosocial well-being, while healthy practices were positively associated with psychosocial well-being and inversely associated with stress/overload. In the adjusted whole-sample models, poorer mental well-being was associated with higher stress/overload and was inversely associated with DQI, physical activity frequency, and family meals. Regular meals were associated with lower odds of both BMI ≥ 25 kg/m^2^ and high stress/overload, whereas highly processed food intake was associated with higher odds of high stress/overload. These findings are consistent with evidence indicating that chronic overload, irregular daily routines, insufficient recovery, and sleep-related difficulties may contribute to less favorable metabolic and emotional functioning [12,13]. They are also compatible with literature linking physical activity with better mental-health indicators and ultra-processed food consumption with poorer mental-health outcomes, although the present cross-sectional data do not allow directionality to be established [11,15,25,26,27].

The gut–brain axis provides a useful conceptual framework for discussing the coexistence of dietary, psychosocial, and stress-related indicators observed in this study. Diet quality, stress exposure, and insufficient recovery may jointly influence microbiota-related, immune, metabolic, and neurobehavioral pathways, thereby linking everyday behaviors with both metabolic and emotional outcomes [16]. However, this mechanistic interpretation must remain cautious. The present study did not include biomarkers of inflammation, gut permeability, microbiota composition, or neuroendocrine regulation. Therefore, the current findings do not demonstrate gut–brain mechanisms directly, but they are compatible with a multidimensional model in which diet quality, stress, recovery, and health-related routines co-occur as interrelated determinants of metabolic and psychological functioning. Recent work in adolescents indicates that pro-inflammatory dietary patterns, including Western-type diets, may be associated with higher inflammatory markers and later depressive symptoms, whereas Mediterranean-like patterns are associated with lower systemic inflammation in this age group [28]. Moreover, studies of depressed adolescents have documented co-occurring gut dysbiosis and elevated pro-inflammatory cytokines, and systematic reviews in youth highlight microbiome disturbances in depression and anxiety, consistent with a gut–brain and immuno-inflammatory pathway linking diet quality and psychosocial distress [17,18,29].

The present results also point to the importance of relational and routine-based factors. Family meals were inversely associated with poorer mental well-being in the adjusted whole-sample model, and regular meals were inversely associated with BMI ≥ 25 kg/m^2^ and high stress/overload. Although these findings cannot be interpreted causally, they are consistent with the broader literature indicating that social connectedness, family support, and supportive relationships are important resources for resilience and long-term health [30,31]. In this context, family meals may reflect not only dietary structure but also regularity, social support, and household-level organization. Similarly, the supplementary stress-and-coping data showed that stress sources and coping strategies differed across age-defined groups, which is compatible with the view that developmental role transitions, socioeconomic context, and time pressure may shape perceived burden and coping style [3,32,33]. These supplementary findings should be interpreted as contextual rather than confirmatory.

Overall, the present findings suggest that health-related functioning across school youth, students, and adults is shaped by the co-occurrence of diet quality, daily practices, psychosocial well-being, stress burden, and behavioral regularity. The results do not support a simple interpretation in which higher knowledge automatically translates into healthier behaviors or better health-related outcomes. Instead, they indicate that behavioral implementation, daily routine, and psychosocial context are important when interpreting the relationship between knowledge and health-related functioning. This interpretation also complements our previous work, in which digital information pathways, especially social media, were more clearly associated with product-oriented behaviors such as supplementation than with overall diet quality itself [19]. From a public health perspective, the findings suggest that future age-sensitive research and prevention strategies should not focus exclusively on education, but should also consider practical support for regular daily routines, physical activity, stress management, recovery-related behaviors, and supportive social environments. Given the cross-sectional, anonymous, self-reported online design and the limited sample size, these implications should be regarded as preliminary and hypothesis-generating.

## 5. Limitations

This study has several limitations. First, its cross-sectional design precludes causal inference and does not allow the direction of the observed associations to be determined. Second, the analyses relied on self-reported online data, including anthropometric, dietary, lifestyle, and psychosocial variables, which may be subject to recall bias, reporting bias, and social desirability bias. Third, although the present analyses were based on the recalculated study sample of 360 respondents, model-specific sample sizes differed because of outcome definitions. In particular, the BMI ≥ 25 kg/m^2^ model was restricted to respondents aged ≥18 years because adult BMI cut-offs were applied, resulting in a model-specific sample size of N = 324. The sample size was sufficient for detecting small-to-moderate whole-sample differences, but not for stable subgroup-specific multivariable modeling. Therefore, subgroup regression models were removed from the final inferential narrative and were not used as a basis for the conclusions. Fourth, the online recruitment strategy may limit the representativeness of the sample and increase the possibility of selection bias. Because the survey was anonymous and web-based, formal drop-out and non-response analyses could not be performed; therefore, non-response bias cannot be excluded. Finally, no biological or clinical markers were collected; therefore, interpretations related to metabolic, inflammatory, or gut–brain mechanisms should be regarded as indirect and hypothesis-generating rather than mechanistically confirmed.

## 6. Conclusions

After narrowing the analytical scope, this cross-sectional online study suggests that selected diet-, lifestyle-, and psychosocial indicators differ across school youth, students, and adults in Poland. The most consistent between-group differences concerned anthropometric status, non-healthy dietary exposure, and selected behavioral indicators, whereas knowledge-related and psychosocial mean differences were limited. In the whole sample, poorer mental well-being and high stress/overload were associated mainly with psychosocial burden, diet quality, processed-food intake, physical activity, and meal-related routines. These findings suggest that behavioral regularity and psychosocial context may be more informative than knowledge alone when describing age-related health profiles. However, because the study was based on anonymous self-reported cross-sectional online data and a modest, non-representative sample, the results should be interpreted as preliminary and hypothesis-generating. Larger prospective studies using validated clinical measures and objective dietary, anthropometric, and biological assessments are needed to confirm these associations and clarify their direction.

## Figures and Tables

**Table 1:** Characteristics and predefined continuous indicators across groups.

Domain	Variable	Overall	School Youth 15–19 y (N = 154)	Students 20–29 y (N = 127)	Adults 30+ y (N = 79)	*p*-Value	Effect Size
Sample	N	360	154	127	79	—	—
	Women, N (%)	229 (63.6)	73 (47.4)	93 (73.2)	63 (79.7)	<0.001	V = 0.296
	Men, N (%)	131 (36.4)	81 (52.6)	34 (26.8)	16 (20.3)	<0.001	V = 0.296
Anthropometry	Age (years), mean ± SD	25.45 ± 10.67	17.97 ± 1.05	23.80 ± 2.83	42.67 ± 9.69	<0.001	η^2^ = 0.792
	Body weight (kg), mean ± SD	71.04 ± 19.44	68.44 ± 14.92	70.65 ± 18.03	76.75 ± 27.04	0.2068	η^2^ = 0.027
	Height (cm), mean ± SD	171.49 ± 10.16	174.08 ± 10.03	170.33 ± 10.51	168.33 ± 8.58	0.0001	η^2^ = 0.054
	BMI (kg/m^2^), mean ± SD	24.06 ± 5.95	22.43 ± 3.69	24.24 ± 5.19	26.94 ± 8.88	<0.001	η^2^ = 0.084
	Waist/hip circumference (cm), mean ± SD	81.74 ± 17.67	75.64 ± 17.15	83.43 ± 14.72	90.92 ± 18.63	<0.001	η^2^ = 0.114
Diet quality	pHDI (0–100), mean ± SD	25.21 ± 14.28	26.41 ± 14.79	24.84 ± 14.08	23.47 ± 13.52	0.3113	η^2^ = 0.007
	nHDI (0–100), mean ± SD	19.44 ± 9.95	21.37 ± 9.20	19.07 ± 9.63	16.27 ± 11.07	<0.001	η^2^ = 0.039
	DQI (−100 to 100), mean ± SD	5.77 ± 15.31	5.04 ± 13.01	5.77 ± 17.46	7.20 ± 15.83	0.4442	η^2^ = 0.003
Knowledge	Obesity knowledge score, mean ± SD	5.28 ± 1.02	5.10 ± 1.20	5.54 ± 0.71	5.20 ± 0.97	0.0011	η^2^ = 0.038
	Depression knowledge score, mean ± SD	4.93 ± 1.17	4.99 ± 1.13	4.91 ± 1.12	4.86 ± 1.33	0.6898	η^2^ = 0.002
	Overall knowledge score, mean ± SD	85.09 ± 15.00	84.09 ± 15.31	87.07 ± 12.80	83.86 ± 17.36	0.1800	η^2^ = 0.010
Practices/psychosocial	Healthy practices score, mean ± SD	56.68 ± 13.58	56.51 ± 11.72	55.95 ± 13.77	58.20 ± 16.42	0.5014	η^2^ = 0.004
	Psychosocial well-being score, mean ± SD	66.97 ± 18.14	67.10 ± 17.59	65.81 ± 20.59	68.57 ± 14.77	0.5682	η^2^ = 0.003
	Stress/overload score, mean ± SD	56.09 ± 17.07	57.52 ± 17.79	55.51 ± 17.00	54.22 ± 15.67	0.3377	η^2^ = 0.006
	Knowledge–practice gap, mean ± SD	0.00 ± 1.26	−0.05 ± 1.28	0.19 ± 1.11	−0.19 ± 1.42	0.0856	η^2^ = 0.014

Values are presented as n (%) or mean ± SD. *p*-values refer to three-group comparisons. η^2^ is reported for continuous variables and Cramér’s V for categorical variables.

**Table 2:** Predefined binary lifestyle and risk outcomes across groups.

Domain	Indicator	Overall	School Youth (N = 154)	Students (N = 127)	Adults (N = 79)	*p*-Value	Cramér’s V
Lifestyle	High or very high self-rated physical activity	96 (26.7%)	58 (37.7%)	34 (26.8%)	4 (5.1%)	<0.001	0.281
	Physical activity ≥ 3 times/week	172 (47.8%)	84 (54.5%)	62 (48.8%)	26 (32.9%)	0.0072	0.166
	Home-prepared meals ≥ 4 times/week	316 (87.8%)	146 (94.8%)	113 (89.0%)	57 (72.2%)	<0.001	0.265
	Highly processed food ≥ 3 times/week	72 (20.0%)	36 (23.4%)	28 (22.0%)	8 (10.1%)	0.0441	0.132
	Sleep < 7 h/night	201 (55.8%)	87 (56.5%)	71 (55.9%)	43 (54.4%)	0.9557	0.016
	Family meals ≥ 4 times/week	160 (44.4%)	74 (48.1%)	45 (35.4%)	41 (51.9%)	0.0339	0.137
Predefined outcomes/risk indicators	BMI ≥ 25 kg/m^2^	116 (32.2%)	30 (19.6%)	47 (37.0%)	39 (49.4%)	<0.001	0.254
	BMI ≥ 30 kg/m^2^	40 (11.1%)	8 (5.2%)	14 (11.0%)	18 (22.8%)	0.0003	0.213
	Poorer mental well-being	86 (23.9%)	44 (28.6%)	33 (26.0%)	9 (11.4%)	0.0114	0.158
	High stress/overload	123 (34.2%)	63 (40.9%)	37 (29.1%)	23 (29.1%)	0.0659	0.123
	Low physical activity	74 (20.6%)	22 (14.3%)	26 (20.5%)	26 (32.9%)	0.0039	0.176
	Poor diet quality (lowest DQI tertile)	121 (33.6%)	59 (38.3%)	38 (29.9%)	24 (30.4%)	0.2632	0.086
	High processed-food intake	72 (20.0%)	36 (23.4%)	28 (22.0%)	8 (10.1%)	0.0441	0.132
	Low fruit/vegetable intake	156 (43.3%)	64 (41.6%)	53 (41.7%)	39 (49.4%)	0.4720	0.065
	Low frequency of family meals	88 (24.4%)	42 (27.3%)	34 (26.8%)	12 (15.2%)	0.0952	0.114
	High knowledge–practice gap	122 (33.9%)	48 (31.2%)	46 (36.2%)	28 (35.4%)	0.6370	0.050

Values are presented as n (%) or mean ± SD. *p*-values refer to three-group comparisons. Cramér’s V for categorical variables.

**Table 3:** Associations among predefined core variables.

Analysis	Variable 1	Variable 2	Coefficient	*p*-Value	q-Value (FDR)
Spearman correlation	Stress/overload score	Psychosocial well-being score	rho = −0.542	6.649 × 10^−29^	3.103 × 10^−28^
	Healthy practices score	Psychosocial well-being score	rho = 0.329	1.617 × 10^−10^	3.234 × 10^−10^
	Healthy practices score	Stress/overload score	rho = −0.273	1.426 × 10^−7^	2.495 × 10^−7^
	Overall knowledge score	Healthy practices score	rho = 0.230	1.091 × 10^−5^	1.696 × 10^−5^
	Diet Quality Index (DQI)	Psychosocial well-being score	rho = 0.164	0.0018	0.0025
	Diet Quality Index (DQI)	Stress/overload score	rho = −0.093	0.0765	0.0823
	BMI (kg/m^2^)	Diet Quality Index (DQI)	rho = 0.064	0.2239	0.2239
	BMI (kg/m^2^)	Healthy practices score	rho = −0.125	0.0178	0.02274
Partial correlation adjusted for age, sex, and group	Diet Quality Index (DQI)	Psychosocial well-being score	partial r = 0.240	4.197 × 10^−6^	1.679 × 10^−5^
	Diet Quality Index (DQI)	Stress/overload score	partial r = −0.127	0.0157	0.0251
	BMI (kg/m^2^)	Healthy practices score	partial r = −0.174	0.0009	0.0024
	BMI (kg/m^2^)	Diet Quality Index (DQI)	partial r = 0.067	0.2082	0.2380
	BMI (kg/m^2^)	Stress/overload score	partial r = −0.084	0.1122	0.1496

**Table 4:** Adjusted models for the three predefined outcomes.

Outcome	Model N	Predictor	Adjusted OR	95% CI	*p*-Value
BMI ≥ 25 kg/m^2^ (respondents aged ≥18 years)	324	Age (years)	1.045	1.019–1.071	0.0005
		Male sex	1.261	0.707–2.249	0.4323
		DQI (per 10 points)	1.367	1.118–1.671	0.0023
		Highly processed food intake	1.241	0.923–1.668	0.1524
		Physical activity frequency	0.727	0.557–0.948	0.0185
		Sleep quality (higher = better)	0.864	0.661–1.129	0.2840
		Stress/overload	0.897	0.781–1.031	0.1253
		Regular meals	0.610	0.478–0.780	0.0001
Poorer mental well-being	360	Age (years)	0.807	0.717–0.907	0.0003
		Male sex	0.671	0.342–1.316	0.2456
		Student status (vs. school youth)	2.793	1.146–6.807	0.0238
		DQI (per 10 points)	0.645	0.502–0.829	0.0006
		Physical activity frequency	0.698	0.511–0.953	0.0238
		Regular meals	1.034	0.775–1.379	0.8223
		Family meals	0.705	0.553–0.898	0.0047
		Sleep quality (higher = better)	0.751	0.542–1.041	0.0856
		Stress/overload	1.389	1.151–1.676	0.0006
High stress/overload	360	Age (years)	0.961	0.913–1.012	0.1331
		Male sex	0.644	0.380–1.090	0.1014
		Student status (vs. school youth)	0.619	0.335–1.145	0.1262
		DQI (per 10 points)	0.902	0.749–1.086	0.2746
		Highly processed food intake	1.325	1.027–1.710	0.0304
		Physical activity frequency	1.273	0.997–1.627	0.0532
		Regular meals	0.689	0.558–0.850	0.0005
		Family meals	1.035	0.845–1.268	0.7372
		Home-prepared meals	0.851	0.612–1.184	0.3388

OR: odds ratio; CI: confidence interval; DQI: Diet Quality Index. Model fit summaries: BMI model N = 324, pseudo R^2^ = 0.125, AIC = 385.72, LLR *p* = 1.312 × 10^−8^; poorer mental well-being model N = 360, pseudo R^2^ = 0.269, AIC = 311.39, LLR *p* = 2.747 × 10^−18^; high stress/overload model N = 360, pseudo R^2^ = 0.075, AIC = 449.51, LLR *p* = 0.0001337.

## Data Availability

The anonymized data underlying this study are available from the corresponding author upon reasonable request. Public deposition was not implemented because the dataset contains detailed health- and behavior-related responses from adolescents and young adults, and additional ethical safeguards were considered necessary to minimize re-identification risk.

## Supplementary Materials

The following supporting information can be downloaded at: https://www.mdpi.com/article/10.3390/nu18122022/s1, Table S1. Questionnaire domains, item coding, score direction, and construction of composite variables; Table S2. Coding of binary indicators and analytical thresholds used in the main analyses; Table S3. Stress sources and coping strategies across school youth, students, and adults; Table S4. Exploratory dietary-pattern loadings and explained variance (A) and exploratory dietary-pattern scores across groups for the selected most interpretable components (B); Table S5. Exploratory cluster distribution across age-defined groups (A) exploratory standardized cluster characteristics (B), and exploratory cluster-validation indices for candidate k-means solutions (C); Figure S1. Exploratory dietary-pattern loadings; Figure S2. Exploratory cluster profile heatmap. Exploratory supplementary analyses were not used as a basis for confirmatory conclusions.
