# Supplementary material for: Diet Quality, Healthy Practices, and Psychosocial Functioning Across School Youth, Students, and Adults in Poland: A Cross-Sectional Online Survey
Source: Nutrients. 2026 Jun 21;18(12):2022. doi: 10.3390/nu18122022 (PMC13304536; doi:10.3390/nu18122022)
Supplement: Supplementary file 1 [file nutrients-18-02022-s001.zip › nutrients-4350298-supplementary.pdf]

Table S1. Questionnaire domains, score direction, and construction of composite variables.

Construct/domain	Raw questionnaire items included	Original response scale / source	Coding and reverse coding	Formula / score construction	Final range / scale	Interpretation and use in revised manuscript
Sociodemographic profile	Age; sex; age-defined group	Direct questionnaire responses	Age was retained as a continuous variable. Age-defined groups were created as follows: school youth, 15–19 years; students, 20–29 years; adults, ≥30 years. Sex was coded as a categorical variable.	Not applicable.	Not applicable.	Used for descriptive comparisons and as covariates in adjusted analyses.
Anthropometry	Self-reported body weight; height; waist/hip circumference	Direct questionnaire responses	Body weight was recorded in kilograms and height in centimeters. Height was converted to meters before BMI calculation.	BMI = body weight (kg) / height² (m²).	Continuous BMI in kg/m²; binary indicators: BMI ≥25 kg/m² and BMI ≥30 kg/m².	BMI ≥25 kg/m² was used as one of the three predefined outcomes in respondents aged ≥18 years. BMI ≥30 kg/m² was used as a descriptive risk indicator.
pHDI — pro-healthy diet index	KomPAN pro-healthy food-frequency items: wholegrain bread; coarse groats/oatmeal/wholegrain pasta; milk; fermented milk beverages; cottage cheese products; white-meat dishes; fish; legume dishes; fruit; vegetables.	KomPAN food-frequency categories: never; 1–3 times/month; once/week; several times/week; once/day; several times/day.	Categories were converted into daily-frequency equivalents according to the KomPAN procedure: never = 0; 1–3 times/month = 0.06; once/week = 0.14; several times/week = 0.5; once/day = 1; several times/day = 2.	pHDI = (sum of daily-frequency values for 10 pro-healthy items / 20) × 100.	0–100.	Higher values indicate more frequent consumption of pro-healthy food groups. Used as a predefined diet-quality indicator.
nHDI — non-healthy diet index	KomPAN non-healthy food-frequency items: refined bread; refined cereal products; fast food; fried dishes; butter; lard; yellow cheese; processed meat products; red meat; sweets; canned meat; sugar-sweetened beverages; energy drinks; alcoholic beverages.	KomPAN food-frequency categories: never; 1–3 times/month; once/week; several times/week; once/day; several times/day.	The same standardized daily-frequency conversion was applied: never = 0; 1–3 times/month = 0.06; once/week = 0.14; several times/week = 0.5; once/day = 1; several times/day = 2.	nHDI = (sum of daily-frequency values for 14 non-healthy items / 28) × 100.	0–100.	Higher values indicate more frequent consumption of less favorable food groups. Used as a predefined diet-quality indicator.
DQI — Diet Quality Index	pHDI and nHDI	Derived from KomPAN-based pHDI and nHDI.	No additional reverse coding was applied beyond the construction of pHDI and nHDI.	DQI = pHDI − nHDI.	Theoretical range: −100 to +100.	Higher values indicate a more favorable balance between pro-healthy and non-healthy food-

Construct/domain	Raw questionnaire items included	Original response scale / source	Coding and reverse coding	Formula / score construction	Final range / scale	Interpretation and use in revised manuscript
						frequency components. Used as the main synthetic diet-quality indicator and as a predictor in adjusted models.
Obesity knowledge score	Six closed obesity-related knowledge items: obesity is a disease; obesity is not merely a feature of appearance; obesity may cause other diseases, including depression; obesity may be fatal; excessive food intake is not the only cause of obesity; moderate physical activity has a beneficial role in obesity management.	Closed yes/no/uncertain knowledge items.	Correct answer = 1; incorrect answer, uncertain answer, or "do not know" = 0. Correct responses were: yes; no; yes; yes; no; yes, respectively.	Obesity knowledge score = sum of correctly answered obesity-related items.	0–6 points.	Higher values indicate greater obesity-related knowledge. Used as a predefined knowledge indicator.
Depression knowledge score	Six closed depression-related knowledge items: knowledge of the nature of depression; moderate physical activity may reduce depressive symptoms; diet may influence mood and emotions; knowledge of consequences of depression; depression may lead to death; correct response to the WHO prevalence-related item.	Closed yes/no/uncertain knowledge items and one multiple-choice prevalence item.	Correct answer = 1; incorrect answer, uncertain answer, or "do not know" = 0. For the item on the nature of depression, both affirmative responses indicating knowledge were coded as correct. For the WHO prevalence item, "280 million people" was coded as correct.	Depression knowledge score = sum of correctly answered depression-related items.	0–6 points.	Higher values indicate greater depression-related knowledge. Used as a predefined knowledge indicator.
Overall knowledge score	Obesity knowledge score and depression knowledge score	Derived from the two knowledge subscores.	The two subscores were summed and transformed to a percentage scale.	Overall knowledge score = [(obesity knowledge score + depression knowledge score) / 12] × 100.	0–100.	Higher values indicate greater overall knowledge related to obesity and depression. Used as a predefined knowledge indicator and as a predictor in selected analyses.
Healthy practices score	Structured items describing health-related practice and routine: self-declared healthy lifestyle; perceived importance of healthy lifestyle; work-related barriers to maintaining a healthy lifestyle; self-rated physical activity;	Agreement, ordinal, and frequency-response items from the author-designed module.	Items were recoded in a favorable direction. Positive items received higher values. Negatively oriented items, including "healthy lifestyle has little importance for my health", work-related barriers to healthy	Each item was recoded to a common favorable-direction metric and transformed to a 0–100 scale. The healthy	0–100.	Higher values indicate more favorable implementation of daily health-related practices and routines. Used as a

Construct/domain	Raw questionnaire items included	Original response scale / source	Coding and reverse coding	Formula / score construction	Final range / scale	Interpretation and use in revised manuscript
	regular meal consumption; eating meals without distractors; frequency of physical activity; frequency of home-prepared meals; daily fruit and vegetable intake; frequency of highly processed food intake; sleep duration; frequency of family meals.		lifestyle, and frequency of highly processed food intake, were reverse-coded. Healthier categories received higher values: more regular meals, meals without distractors, higher physical activity, more frequent home-prepared meals, higher fruit/vegetable intake, lower processed-food intake, adequate sleep duration, and more frequent family meals.	practices score was calculated as the mean of the recoded item scores.		predefined behavioral indicator and for calculation of the knowledge–practice gap.
Psychosocial well-being score	Structured items describing psychosocial functioning: satisfaction with contact with the closest family; self-rated general health; self-rated mental well-being; ability to cope with difficult emotions; ability to cope with stress; negative influence of work on well-being.	Binary, ordinal, and agreement-type items from the author-designed module.	Items were coded so that higher values indicated more favorable psychosocial functioning. Negatively oriented items, including negative influence of work on well-being, were reverse-coded.	Each item was recoded to a common favorable-direction metric and transformed to a 0–100 scale. The psychosocial well-being score was calculated as the mean of the recoded item scores.	0–100.	Higher values indicate better psychosocial well-being. Used as a predefined psychosocial indicator and to define poorer mental well-being for the main binary outcome.
Poorer mental well-being	Psychosocial well-being score	Derived binary variable.	Participants in the lower/unsatisfactory range of the psychosocial well-being score were coded as 1; all others were coded as 0.	Binary indicator based on the predefined lower range of psychosocial well-being.	0/1.	Used as one of the three predefined outcomes in the main analyses.
Stress/overload score	Four stress/overload items: sleeping too little and waking tired; intensive work/feeling overworked or overstimulated by tasks; finding time for oneself/relaxation; inability to cope with stress or emotional discharge.	Frequency-response items: not at all; several times/year; several times/month; daily or almost daily.	Adverse items were coded in increasing burden direction: not at all = 0; several times/year = 1; several times/month = 2; daily or almost daily = 3. The relaxation item was reverse-coded so that lower frequency of relaxation contributed to higher burden.	Stress/overload score = (sum of coded item values / 12) × 100.	0–100.	Higher values indicate greater perceived stress/overload burden. Used as a predefined psychosocial indicator and to define high stress/overload.
High stress/overload	Stress/overload score	Derived binary variable.	Participants in the upper tertile of the stress/overload score distribution were coded as 1; all others were coded as 0.	Binary indicator based on the upper tertile of the stress/overload distribution.	0/1.	Used as one of the three predefined outcomes in the main analyses.

Construct/domain	Raw questionnaire items included	Original response scale / source	Coding and reverse coding	Formula / score construction	Final range / scale	Interpretation and use in revised manuscript
Knowledge–practice gap	Overall knowledge score and healthy practices score	Derived from standardized knowledge and practice scores.	Overall knowledge and healthy practices scores were standardized before comparison.	Knowledge–practice gap = z(overall knowledge score) − z(healthy practices score).	Continuous standardized difference score.	Higher values indicate a greater discrepancy between declared knowledge and reported implementation of healthy practices. Reported descriptively; not used as a central inferential outcome.
Stress sources	Work/job demands; family/personal problems; financial problems; school/exam pressures; health-related stress.	Multiple-choice item.	Each selected stress source was coded as 1; each non-selected source was coded as 0.	Separate binary indicators were created for each stress source.	0/1 for each source.	Presented as supplementary contextual information only.
Coping strategies	Spending time with family/friends; physical exercise; meditation/yoga; music/reading/relaxation; sleep/rest; professional support; non-adaptive coping.	Multiple-choice item.	Each selected coping strategy was coded as 1; each non-selected coping strategy was coded as 0.	Separate binary indicators were created for each coping strategy.	0/1 for each strategy.	Presented as supplementary contextual information only.

pHDI, nHDI, and DQI were calculated using KomPAN-based food-frequency conversion rules. Eggs were included in the food-frequency module but were not included in the pHDI calculation. Open-ended items, including descriptions of depressive symptoms, perceived causes of obesity, or definitions of mental health, were not used for knowledge-score calculation because they were not uniformly coded as closed correct/incorrect responses.

Table S2. Coding of binary indicators and analytical thresholds used in the main analyses.

Binary variable	Definition/threshold	Role after revision
BMI ≥25 kg/m²	BMI calculated from self-reported weight and height; coded 1 if BMI ≥25 kg/m².	Primary predefined outcome in respondents aged ≥18 years.
BMI ≥30 kg/m²	Coded 1 if BMI ≥30 kg/m².	Descriptive risk indicator.
Poorer mental well-being	Binary category based on the lower/unsatisfactory range of the psychosocial well-being score.	Primary predefined outcome.
High stress/overload	Upper tertile of the stress/overload distribution.	Primary predefined outcome.
Short sleep	Sleep duration <7 h/night.	Descriptive binary indicator.
Low physical activity	Low category of physical activity according to survey coding.	Descriptive risk indicator; predictor in selected models.
High processed-food intake	Highly processed food intake ≥3 times/week.	Descriptive indicator and model predictor.
Poor diet quality	Lowest tertile of DQI.	Descriptive risk indicator only.
Low fruit/vegetable intake	Low intake category based on food-frequency coding.	Descriptive risk indicator only.

Binary variable	Definition/threshold	Role after revision
Low family-meal frequency	Low frequency category of family meals.	Descriptive risk indicator only.
High knowledge–practice gap	Upper category of the knowledge–practice gap indicator.	Descriptive indicator only.

Table S3. Stress sources and coping strategies across age-defined groups.

Variable	School youth	Students	Adults	p-value	Cramér's V
Work/job demands	117 (76.0%)	108 (85.0%)	52 (65.8%)	0.0059	0.169
Family/personal problems	75 (48.7%)	65 (51.2%)	37 (46.8%)	0.8223	0.033
Financial problems	28 (18.2%)	32 (25.2%)	12 (15.2%)	0.1649	0.100
School/exam pressures	12 (7.8%)	0 (0.0%)	0 (0.0%)	0.0002	0.215
Health-related stress	2 (1.3%)	0 (0.0%)	0 (0.0%)	0.2605	0.086
Time with friends/family	102 (66.2%)	81 (63.8%)	67 (84.8%)	0.0032	0.178
Physical exercise	79 (51.3%)	48 (37.8%)	26 (32.9%)	0.0111	0.158
Meditation/yoga	25 (16.2%)	22 (17.3%)	12 (15.2%)	0.9202	0.022
Music/reading/relaxation	8 (5.2%)	8 (6.3%)	0 (0.0%)	0.0860	0.117
Sleep/rest	0 (0.0%)	2 (1.6%)	2 (2.5%)	0.1800	0.098
Professional support	2 (1.3%)	2 (1.6%)	0 (0.0%)	0.5528	0.057

Table S4A. Exploratory dietary-pattern loadings and explained variance.

Component	Exploratory pattern name	Strongest loadings	Eigenvalue	% variance
F1	Dairy + meat products pattern	Cottage cheese products (0.699); fermented milk beverages (0.656); white-meat dishes (0.641); red-meat dishes (0.545); processed meat products (0.537)	4.671	18.632
F2	Coarse groats, fruit and vegetables pattern	Coarse groats/oatmeal/wholegrain pasta (0.553); sugar-sweetened beverages (−0.528); vegetables (0.488); fruit (0.479); energy drinks (−0.426)	2.723	10.861
F3	Legumes, canned meat, lard and fish pattern	Legume dishes (0.683); canned meat (0.667); lard (0.563); fish (0.540); refined bread (−0.438)	2.361	9.416
F4	Sweetened beverages, energy drinks and alcohol pattern	Energy drinks (0.614); alcoholic beverages (0.580); sugar-sweetened beverages (0.500); butter (−0.364); legume dishes (−0.254)	1.561	6.227
F5	Sweets, alcohol and butter pattern	Sweets (0.492); yellow cheese (−0.481); alcoholic beverages (0.416); butter (0.369); processed meat products (−0.320)	1.328	5.296

Table S4B. Exploratory dietary-pattern scores across groups.

Pattern score	School youth mean ± SD	Students mean ± SD	Adults mean ± SD	ANOVA p
Pattern 1 (18.6%)	0.36 ± 2.46	−0.05 ± 1.75	−0.63 ± 2.00	0.0038
Pattern 2 (10.9%)	−0.23 ± 1.30	0.04 ± 1.89	0.38 ± 1.80	0.0277
Pattern 3 (9.4%)	0.05 ± 1.57	−0.09 ± 1.36	0.05 ± 1.75	0.7160

Note: Although five exploratory PCA components were retained and are reported in Table S4A, Table S4B presents between-group comparisons only for the first three components because these components had the clearest interpretability and were considered the most relevant for supplementary descriptive comparison. Components F4 and F5 are reported in the loading table but were not used for between-group interpretation. PCA was performed on standardized KomPAN food-frequency variables using principal

component extraction. The overall KMO value was 0.699, and Bartlett's test of sphericity was significant (χ^2 = 2930.20, df = 300, p < 0.001). The five retained supplementary components explained 50.43% of the total variance. Loadings with absolute values ≥0.30 were considered informative. PCA was retained only as exploratory supplementary material and was not used as a basis for confirmatory conclusions. Between-group comparisons were presented only for selected components with the clearest descriptive interpretability.

Table S5A. Exploratory cluster distribution across age-defined groups.

Cluster/profile	N	School youth	Students	Adults
Cluster 1: High stress / lower well-being	144	74 (48.1%)	45 (35.4%)	25 (31.6%)
Cluster 2: Higher BMI / poorer practices	51	25 (16.2%)	16 (12.6%)	10 (12.7%)
Cluster 3: Healthier profile	164	54 (35.1%)	66 (52.0%)	44 (55.7%)

Table S5B. Exploratory standardized cluster characteristics.

Cluster/profile	BMI z	DQI z	Knowledge z	Practices z	Stress z	Well-being z
Cluster 1: High stress / lower well-being	−0.05	−0.39	0.20	−0.51	0.82	−0.72
Cluster 2: Higher BMI / poorer practices	0.48	−0.45	−1.88	−0.52	−0.50	0.34
Cluster 3: Healthier profile	−0.11	0.48	0.41	0.61	−0.56	0.53

Notes for Table S5: Cluster analysis was performed using k-means clustering with Euclidean distance on z-standardized BMI, DQI, overall knowledge, healthy practices, stress/overload, and psychosocial well-being. Candidate solutions from k = 2 to k = 6 were compared using interpretability, cluster-size distribution, within-cluster sum of squares, silhouette coefficient, Calinski–Harabasz index, and Davies–Bouldin index. The three-cluster solution was retained as an interpretable exploratory solution. Cluster analysis should not be interpreted as a confirmatory classification.

Table S5C. Exploratory cluster-validation indices for candidate k-means solutions.

k	Silhouette coefficient	Calinski–Harabasz index	Davies–Bouldin index	Within-cluster sum of squares
2	0.196	90.3	1.801	1719.2
3	0.194	81.6	1.632	1477.0
4	0.157	71.9	1.681	1340.0
5	0.176	69.1	1.533	1209.2
6	0.162	65.6	1.540	1116.4

Candidate solutions from k = 2 to k = 6 were compared. The three-cluster solution was retained because it provided interpretable, non-trivial profiles with acceptable cluster-size distribution and improved Davies–Bouldin index compared with the two-cluster solution. Cluster analysis was exploratory and was not used for confirmatory inference.

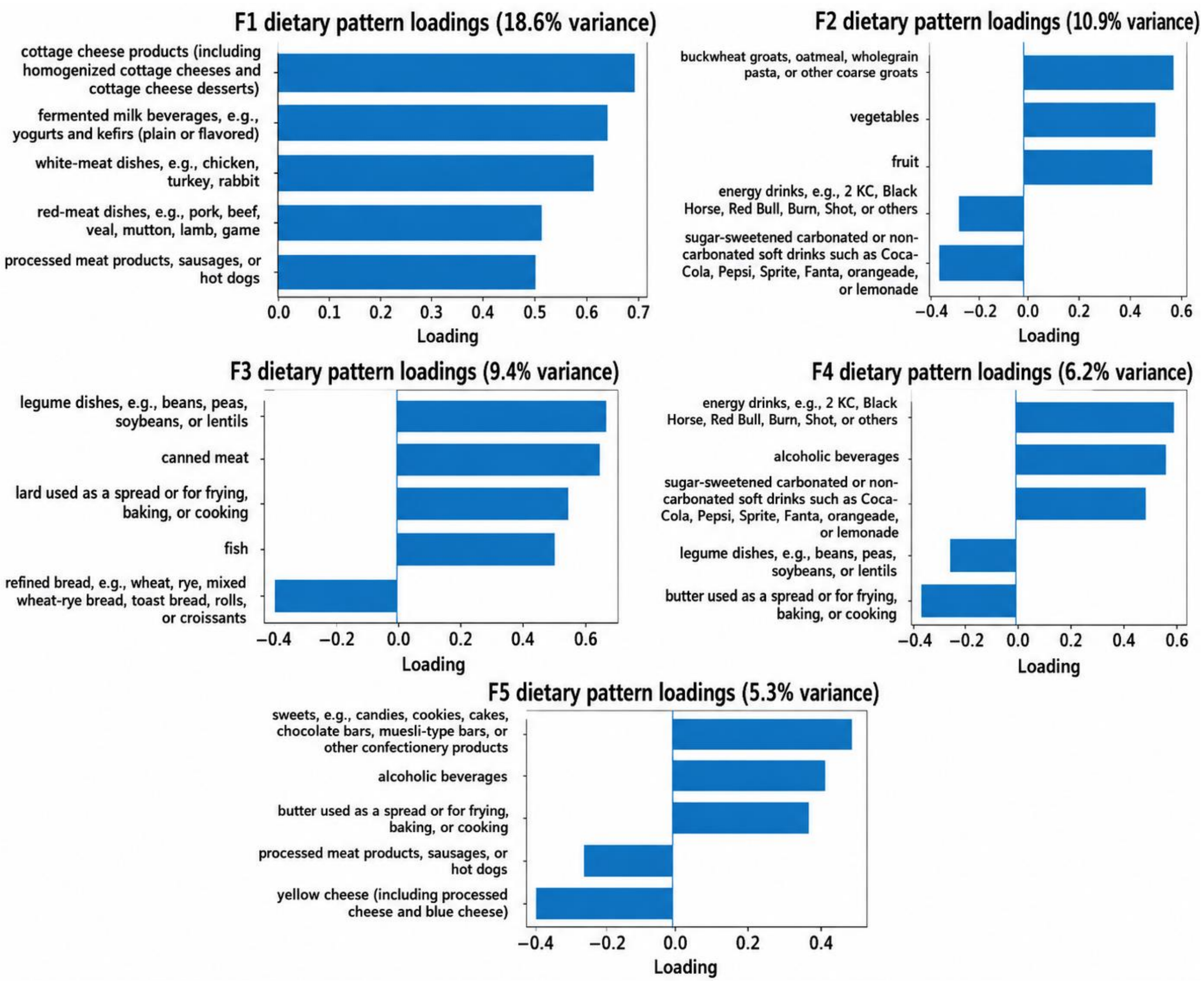
F1 dietary pattern loadings (18.6% variance)
cottage cheese products (including homogenized cottage cheeses and cottage cheese desserts)
fermented milk beverages, e.g., yogurts and kefirs (plain or flavored)
white-meat dishes, e.g., chicken, turkey, rabbit
red-meat dishes, e.g., pork, beef, veal, mutton, lamb, game
processed meat products, sausages, or hot dogs
Loading
F2 dietary pattern loadings (10.9% variance)
buckwheat groats, oatmeal, wholegrain pasta, or other coarse groats
vegetables
fruit
energy drinks, e.g., 2 KC, Black Horse, Red Bull, Burn, Shot, or others
sugar-sweetened carbonated or non-carbonated soft drinks such as Coca-Cola, Pepsi, Sprite, Fanta, orangeade, or lemonade
Loading
F3 dietary pattern loadings (9.4% variance)
legume dishes, e.g., beans, peas, soybeans, or lentils
canned meat
lard used as a spread or for frying, baking, or cooking
fish
refined bread, e.g., wheat, rye, mixed wheat-rye bread, toast bread, rolls, or croissants
Loading
F4 dietary pattern loadings (6.2% variance)
energy drinks, e.g., 2 KC, Black Horse, Red Bull, Burn, Shot, or others
alcoholic beverages
sugar-sweetened carbonated or non-carbonated soft drinks such as Coca-Cola, Pepsi, Sprite, Fanta, orangeade, or lemonade
legume dishes, e.g., beans, peas, soybeans, or lentils
butter used as a spread or for frying, baking, or cooking
Loading
F5 dietary pattern loadings (5.3% variance)
sweets, e.g., candies, cookies, cakes, chocolate bars, muesli-type bars, or other confectionery products
alcoholic beverages
butter used as a spread or for frying, baking, or cooking
processed meat products, sausages, or hot dogs
yellow cheese (including processed cheese and blue cheese)
Loading

Figure S1. Exploratory dietary-pattern loadings.

Loadings are shown for retained exploratory dietary components derived from standardized KomPAN food-frequency variables. Loadings with absolute values ≥0.30 were considered informative. PCA was retained only as supplementary exploratory material and was not used as a basis for confirmatory conclusions. DQI: Diet Quality Index; pHDI: pro-healthy diet index; nHDI: non-healthy diet index.

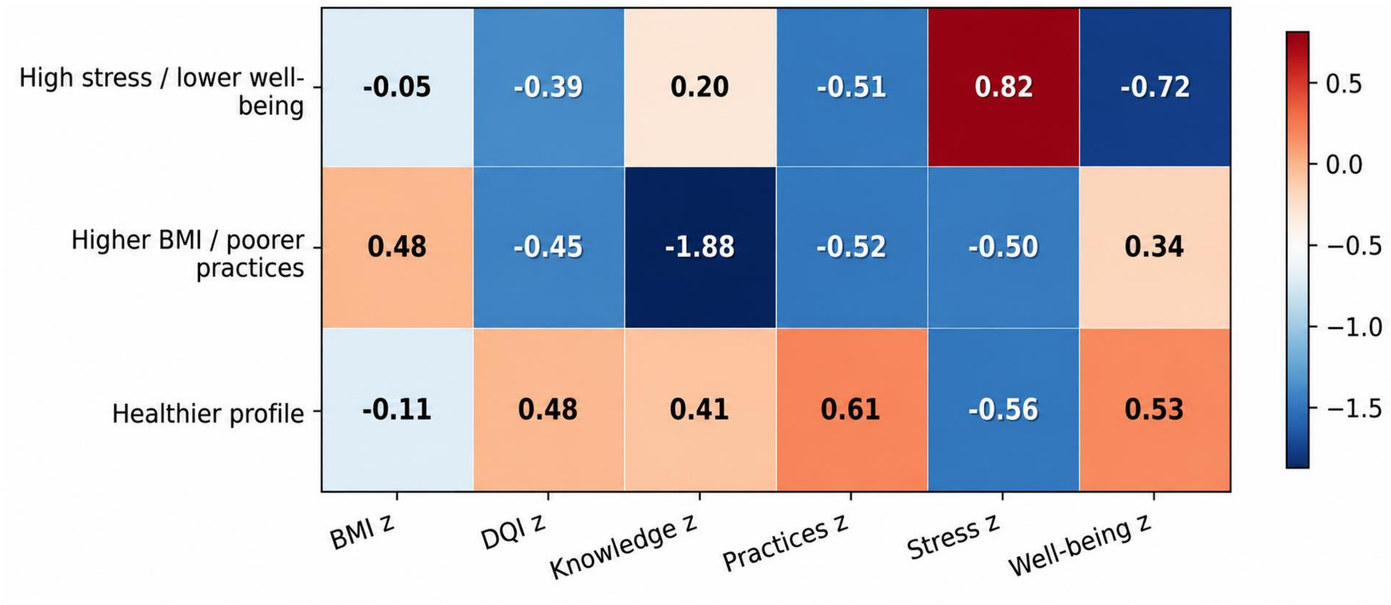

Figure S2. Exploratory cluster profile heatmap.

Standardized values illustrate exploratory cluster profiles across BMI, DQI, overall knowledge, healthy practices, stress/overload, and psychosocial well-being. Cluster analysis was performed using k-means clustering with Euclidean distance on z-standardized variables. The three-cluster solution was retained for supplementary descriptive purposes only and should not be interpreted as a confirmatory classification.
